# Study on the Calcium Transport-Promoting Property and Mechanism of the Peptide–Calcium Complex DEEENDQVK–Ca Based on a Caco-2 Monolayer Model

**DOI:** 10.3390/foods14173119

**Published:** 2025-09-06

**Authors:** Yaxin Zhang, Jingjing Ru, Shan Gao, Hongli Zhi, Wei Zhao, Chunyan Hao, Xiaowei Zhang

**Affiliations:** Tianjin Key Laboratory of Food Quality and Health, College of Food Science and Engineering, Tianjin University of Science & Technology, Tianjin 300457, China; z5265212022@163.com (Y.Z.);

**Keywords:** peptide–calcium complex, calcium transport channel, molecular docking, TRPV6

## Abstract

Peptide–calcium complexes exhibit promise as calcium supplements due to their enhanced bioavailability. Phosvitin nonapeptide DEEENDQVK (DK) possesses a high calcium-binding capability. This study investigated the calcium transport-promoting properties of DK and DEEENDQVK–calcium complex (DK–Ca) using a Caco-2 monolayer model. Both DK and DK–Ca concentration-dependently promoted calcium transport, and compared with the CaCl_2_ control, enhanced calcium transport by 1.07-fold and 1.83-fold, respectively. The calcium transport channels and mechanisms of DK and DK–Ca were also investigated using transfer channel regulators, real-time quantitative polymerase chain reaction, and molecular docking. The results showed that DK–Ca primarily promoted calcium transport through the TRPV6 channel, with supplementary contributions from paracellular and endocytosis channels, while DK did not rely on the endocytosis channel. DK and DK–Ca promoted calcium transport by upregulating TRPV6, calbindin-D9k, PMCA1b, and claudin-2 mRNA expression. DK–Ca exhibited a higher binding affinity for TRPV6 (−10.0 kcal/mol) compared to DK (−5.5 kcal/mol). DK–Ca primarily binds TRPV6′s extracellular exposed cavity through hydrogen bonds and hydrophobic interactions, increasing the local calcium concentration at the channel entrance to promote calcium absorption. This study provides cellular-level mechanistic clues for the potential effects of DK–Ca as a calcium supplement.

## 1. Introduction

Calcium represents an essential mineral that is vital for human structural formation and physiological homeostasis [1]. Approximately 99% of calcium is deposited in the skeleton and teeth as calcium phosphate, a compound that promotes bone strength and provides critical structural support to hard tissues [2]. Additionally, calcium is integral to diverse physiological processes, including bone growth, nerve signal transmission, muscle contraction, and blood coagulation [3]. Calcium deficiency not only contributes to skeletal disorders such as rickets, osteomalacia, and osteoporosis but is also associated with systemic health risks, including hypertension, pregnancy complications, colorectal cancer, obesity, and cardiovascular diseases [4,5].

Dietary calcium supplementation offers a direct solution to calcium deficiency. However, traditional calcium supplements (e.g., calcium carbonate) suffer from limitations such as low bioavailability and gastrointestinal irritation, which lead to reduced efficacy [6]. Research has proved that combining bioactive peptides derived from dietary proteins with calcium can enhance calcium absorption [7]. Compared to traditional calcium supplements, peptide–calcium complexes have superior calcium absorption and bioavailability [8]. This enhanced performance is primarily attributed to the peptides’ high calcium-binding capacity. Furthermore, these complexes protect calcium from interactions with dietary inhibitors (e.g., phytic acid, oxalic acid) within the intestine, thereby preventing precipitation and facilitating absorption [9,10]. Consequently, peptide–calcium complexes have become a major research focus in calcium supplementation. Wang et al. [11] extracted collagen peptides rich in free calcium from sheep bones, reaching a calcium-binding capacity of 42.57 mg/g. Wang et al. [12] reported that CPP had a calcium-binding capacity of 60.17 mg/g. Liao et al. [13] isolated and identified a novel casein calcium-binding peptide (VLPVPQK), exhibiting a calcium-binding capacity of 129.46 mg/g. Liu [14] identified two *tilapia* calcium-binding peptides, YGTGL and LVFL, with binding capacities of 76.03 mg/g and 79.50 mg/g, respectively. It is still necessary to discover peptides with higher calcium-binding capacities. Phosvitin in egg yolk is a naturally evolved carrier of calcium and phosphate, playing a crucial role in chicken embryonic development [15]. The phosphorus required for bone mineralization in chicken embryos is entirely supplied by phosvitin from the egg yolk [15]. Furthermore, phosvitin exhibits a high calcium-binding capacity, which is the key to its ability to steadily promote bone development [16]. Our previous study isolated and identified a nonapeptide, DEEENDQVK (DK), from phosvitin. DK contains a continuous acidic amino acid sequence, which is a structural hallmark of phosvitin and ensures its high mineral binding capacity, and has a calcium binding capacity of 151.1 mg/g [17]. It is currently the active peptide with the highest calcium-binding capacity that has been reported to have been prepared through separation [17]. However, the unclear calcium transport-promoting properties and mechanisms of peptide–calcium complexes limit their commercial applications.

The Caco-2 monolayer, a well-established model of human intestinal epithelial cells, is extensively employed in nutrient absorption studies, including studies on protein hydrolysates, bioactive peptides, and minerals [10,18,19]. For example, Liao et al. [20] demonstrated that three novel *tilapia collagen*-derived peptides (GPAGPHGPVG, FDHIVY, and YQEPVIAPKL) significantly promoted intestinal calcium transport within 30 min, with increases of 89%, 202%, and 130%, respectively, compared with the control group. Wang et al. [21] identified a new peanut calcium-binding peptide (FPPDVA), and the FPPDVA–calcium complex promoted calcium absorption via the Cav1.3 and TRPV6 calcium channels. Li et al. [22] discovered that both casein phosphopeptides (CPPs) and CPP–calcium promoted calcium absorption by upregulating the TRPV5 and TRPV6 genes and protein expression, increasing calcium transport by 77% and 129%, respectively, compared to CaCl_2_. Similar to CPP, phosvitin peptides with a high calcium binding capacity had the potential to promote calcium absorption. Through mouse animal model and Caco-2 monolayer model experiments, Zhao et al. [23] demonstrated that phosvitin peptide–calcium complexes enhanced intestinal calcium absorption by regulating calbindin-D9k expression within the TRPV6 calcium transport channel, exhibiting a 0.85-fold higher calcium transport capacity than the CaCl_2_ control group. However, it is difficult to clarify the specific calcium absorption properties and mechanisms of the peptides in the above study because they are mixtures. Zhang et al. [17] studied the stability of peptide–calcium chelates through in vitro simulation of gastrointestinal digestion. After digestion for 4 h, the solubility of the DK–Ca was 1.56 times that of the blank control group (CaCO_3_). Additionally, as the digestion process progressed, the calcium dialysis rate of DK–Ca was the highest, reaching 1.79 times that of CaCO_3_. These results indicated that DK–Ca remained highly stable and exhibited superior bioavailability after gastrointestinal digestion. However, the calcium transport-promoting properties and mechanisms of DK and DK–Ca remain unclear. Thus, in this study, the calcium transport-promoting properties of DK and DK–Ca were investigated through the Caco-2 monolayer model and compared to CaCl_2_. In addition, the calcium transport mechanisms were clarified using real-time quantitative polymerase chain reaction (RT-qPCR) analysis and molecular docking technology. This study will help to better demonstrate the potential application prospects of DK–Ca in promoting calcium absorption at the cellular level.

## 2. Materials and Methods

### 2.1. Materials and Reagents

Caco-2 cells were obtained from the Shanghai Cell Bank of the Chinese Academy of Sciences (Shanghai, China). DEEENDQVK (purity >98% by HPLC, 1106.2 Da) was synthesized using the Fmoc solid-phase peptide synthesis method and purified by HPLC at Hefei Sener Biological Co., Ltd. (Anhui, China). MTT, dimethyl sulfoxide (DMSO), and 0.25% pancreatic enzyme (including 0.02% EDTA) were purchased from Beijing Solarbio Technology Co., Ltd. (Beijing, China). Dulbecco’s Modified Eagle’s Medium (DMEM) was purchased from Gibco (New York, NY, USA). Fetal bovine serum (FBS) was purchased from ExCell Bio Group (Shanghai, China). Calcium colorimetric assay kit was purchased from Shanghai Biyuntian Biotechnology Co., Ltd. (Shanghai, China). 2-Aminoethoxy diphenylborate (2-APB) was purchased from Tianjin Qianhui Technology Co., Ltd. (Tianjin, China). Cytochalasin D was purchased from Shanghai Yuanye Biotechnology Co., Ltd. (Shanghai, China). Methyl-β-cyclodextrin (mβCD) and casein phosphopeptides (CPP) were purchased from Shanghai Macklin Biochemical Co., Ltd. (Shanghai, China). Fluo-4 AM was purchased from Yisheng Biotechnology Co., Ltd. (Shanghai, China). RNAiso Plus and PrimeScript^®^ RT reagent kit were purchased from Takara Bio Inc. (Dalian, China). All other chemicals and solvents employed were of analytical quality.

### 2.2. Preparation of DK–Ca

DK–Ca was synthesized according to the method of Zhang et al. [17]. DK (1 mg/mL) was dissolved in a 20 mM Tris-HCl buffer at pH 7.8. Anhydrous CaCl_2_ was subsequently introduced at a DK/calcium molar ratio of 1:50 (g/mmol). The mixture was stirred at 50 °C for 1 h. Following that, nine times the volume of anhydrous ethanol was added, and after letting it sit for 2 h, the mixture was centrifuged at 10,000× *g* for 15 min. The precipitate was collected and lyophilized to obtain DK–Ca. Calcium-binding capacity was quantified using a calcium assay kit with the o-cresol phthalein complexone method [24]. Three independent experiments demonstrated that DK exhibited a calcium-binding capacity of 150 ± 4.73 mg/g; this result aligns with the previous study [17].

### 2.3. Caco-2 Cell Culture and Cell Viability Assays

Caco-2 cells were maintained in complete DMEM medium containing 20% (*v*/*v*) FBS, 1% non-essential amino acids, and penicillin-streptomycin (100 U/mL−100 μg/mL) at 37 °C under 5% CO_2_ atmosphere, with medium replacement every 48 h. At 80–90% confluency, cells were seeded into 96-well plates (1 × 10^4^ cells/well). After 24 h of incubation, the original medium was exchanged for a new solution containing CaCl_2_ (0~5 mg/mL), DK (0~5 mg/mL), DK + CaCl_2_ (1:1 *w*/*w*, 0~5 mg/mL), DK–Ca (0~5 mg/mL), 2-APB (0~75 μmol/L), cytochalasin D (0~2 μg/mL), and mβCD (0~5 mmol/L). After 24 h of incubation, the cell viability was assessed by MTT [21].

### 2.4. Establishment of Caco-2 Monolayer Model

Caco-2 cells (passages 15–30) were seeded at 1 × 10^5^ cells/mL in Transwell chambers (12 mm, 0.4 μm pores, polycarbonate membrane, 1.12 cm^2^ area) to establish a Caco-2 monolayer model. The culture medium (0.5 mL in the apical and 1.5 mL in the basolateral) was refreshed every 48 h (first week), then daily (next two weeks). The integrity of the Caco-2 cell monolayer model was verified by the following multiple indicators: Transepithelial electrical resistance (TEER) was monitored every 48 h using an epithelial voltohmmeter (RE1600, Beijing Jingong Hongtai Technology Co., Ltd., Beijing, China). Alkaline phosphatase (ALP) activity was measured on days 8, 12, 14, 16, and 21 after inoculation utilizing an ALP assay kit, following the manufacturer’s protocol. Transepithelial permeability was evaluated using sodium fluorescein following the method of Qiu et al. [25].

### 2.5. Calcium Transport Assay

Caco-2 monolayer models with a TEER value exceeding 500 Ω·cm^2^ are permitted for calcium transport studies [26]. Following three sequential washes with Hank’s balanced salt solution (HBSS; calcium/magnesium-free, pH 7.4), monolayers were equilibrated at 37 °C for 30 min. Afterward, the Transwell chambers were moved to fresh receiver plates for transport assays.

Concentration-dependent CaCl_2_ transport assay: 0.5 mL of CaCl_2_ solution (1.5~10 mM) was added to the apical (AP) side. The basolateral (BL) side contained 1.5 mL prewarmed HBSS. After 120 min incubation at 37 °C and 5% CO_2_, the BL side solution was collected for calcium transport analysis.

Time-dependent CaCl_2_ transport assay: The AP side contained 0.5 mL of 7.5 mM CaCl_2_ solution, and the BL side 1.5 mL prewarmed HBSS. At designated intervals (30~180 min), 1 mL BL side solution was collected and replaced with equal volumes of fresh HBSS buffer to maintain consistency.

Different concentrations of DK solution (0.5~2 mg/mL, 0.5 mL) containing 7.5 mM CaCl_2_, or 0.5 mL of DK–Ca solution (0.5~2 mg/mL), were added to the AP side. The BL side contained 1.5 mL prewarmed HBSS. The control group contained 7.5 mM CaCl_2_ alone on the AP side. At designated intervals (30~180 min), 1 mL BL side solution was collected and replaced with equal volumes of fresh HBSS buffer to maintain consistency.

Calcium concentration in the collected sample was measured using a calcium colorimetric assay kit at 575 nm [27]. Transported calcium was calculated as follows:

(1) $$T_{n}=1.5C_{n}+1\times \sum_{k=1}^{n-1}C_{k}$$

*T_n_* represents the total accumulation of transported calcium on the BL side at each sampling time point (μg/well); 1.5 represents the volume of HBSS buffer in the BL (mL); *C_n_* represents the calcium concentration on the BL side at the corresponding time point (μg/mL); 1 denotes the 1 mL of sample solution collected from the BL; *n* represents different time points, specifically 1, 2, 3, 4, or 5, corresponding to 30, 60, 90, 120, and 180 min. *C_k_* represents the transported calcium at time point *k*.

### 2.6. Effects of Different Samples and Dietary Components on Calcium Transport

Each sample with a calcium content of 300 μg/mL (0.5 mL) was added to the AP side: 7.5 mM CaCl_2_, 2 mg/mL CPP + 7.5 mM CaCl_2_, 2 mg/mL DK + 7.5 mM CaCl_2_, and 2 mg/mL DK–Ca. The BL side contained 1.5 mL prewarmed HBSS buffer. After 120 min incubation at 37 °C and 5% CO_2_, the BL side solution was collected for calcium transport analysis.

Vitamin D3 (0.5 μmol/L, dissolved in 0.1% ethanol) or phytic acid (1.8 mM, with a of phytic acid/calcium molar ratio of 0.24) was added to the AP side to evaluate the effect of dietary components on calcium transport. The group without an added dietary component served as the control group. After 120 min incubation, the BL side solution was collected for calcium transport analysis.

### 2.7. Component Detection After DK–Ca Transmembrane Transport

The AP side received 2 mg/mL DK–Ca solution while the BL side contained 1.5 mL prewarmed HBSS. Following incubation for 120 min at 37 °C and 5% CO_2_, solutions from both sides were analyzed for their component changes by reversed-phase high-performance liquid chromatography (RP-HPLC, Agilent1260, Agilent Technologies Inc., Santa Clara, CA, USA) [28]. Chromatographic separation occurred on a C18 column (250 mm × 4.6 mm, ACE Excel 5 SuperC18, Avantor, UK) with mobile phase A (0.1% trifluoroacetic acid in water) and mobile phase B (0.1% trifluoroacetic acid in acetonitrile). The gradient elution was programmed as follows: 0~30 min, 5% to 35% B, and 30~33 min, 35% to 100% B. The flow rate was set at 0.8 mL/min with a 30 μL injection volume. Detection was monitored at 207 nm [17]. The raw DK–Ca solution before the transport experiment was used as a control.

### 2.8. Visual Detection of Calcium Transport-Promoting Effects

Fluorescence imaging based on intracellular calcium influx was conducted to assess the calcium transport-promoting effect in the Caco-2 monolayer following the method of Men et al. [29]. Log-phase cells were seeded at 1.5 × 10^5^ cells/well in 35 mm laser confocal dishes and gently agitated to ensure uniform distribution. Cultures were maintained at 37 °C in 5% CO_2_ with daily medium replacement until confluent monolayers formed. Subsequently, CaCl_2_, DK + CaCl_2_, and DK–Ca solutions (each containing 150 μg/mL calcium) were added separately. The group without an added sample served as the control group. Following 24 h of incubation, the cells were washed thrice with prewarmed calcium-/magnesium-free HBSS buffer (pH 7.2). Under light-protected conditions, 200 µL of Fluo-4 AM (4 μmol/L) was applied to cover the cells. Following 30 min incubation at 37 °C and subsequent washes, cells were dark-adapted for 10 min at room temperature for de-esterification. Imaging was conducted using a confocal microscope (excitation at 496 nm, Zeiss LSM980 Airyscan2, Carl Zeiss, Germany ), followed by quantification of the fluorescence intensity with ImageJ 1.54 g software.

### 2.9. Calcium Transport Channel Assay

Before the calcium transport channel assay, 0.5 mL of HBSS buffer containing different channel regulators was added to the AP sides of the Transwell chambers. These channel regulators included 2-APB (25 μmol/L) as a calcium channel inhibitor of TRPV6, cytochalasin D (0.5 μmol/ mL) as a paracellular channel promoter, and mβCD (3 mmol/L) as an endocytic channel inhibitor. The group without the added channel regulator served as the control group. The BL side received a 1.5 mL HBSS buffer. Following 30 min incubation, solutions were removed from both sides. The AP side was then loaded with 0.5 mL of either DK–Ca (2 mg/mL) or 2 mg/mL DK + 7.5 mM CaCl_2_ solution, while the BL side received 1.5 mL HBSS. After 120 min incubation at 37 °C and 5% CO_2_, the BL solution was collected for calcium transport analysis.

### 2.10. Real-Time Quantitative Polymerase Chain Reaction (RT-qPCR)

The expression levels of genes related to calcium transport channels were determined following the method of Li et al. [22]. After 21 days of establishing the Caco-2 monolayer model, the monolayers were treated for 24 h with a complete medium containing 2 mg/mL DK–Ca or 2 mg/mL DK + 7.5 mM CaCl_2_. The group without an added sample served as the control group. Subsequently, the monolayers were washed thrice with HBSS buffer before cell lysis using 200 μL RNAiso Plus. Total RNA was extracted, and first-strand cDNA synthesis was subsequently performed with the PrimeScript^®^ RT reagent kit as per the kit manufacturer’s instructions. Using cDNA as a template, RT-qPCR was performed with gene-specific primers targeting TRPV6, calbindin-D9k, PMCA1b, occludin, and claudin-2. The standard procedure for PCR amplification was as follows: 95 °C for 30 s, followed by 40 cycles of 95 °C for 5 s, 60 °C for 30 s, and 72 °C for 1 min. Target gene expression level was normalized to GAPDH and calculated using the 2^^(-ΔΔCt)^ relative quantification method. In experimental groups, GAPDH served as the internal reference gene for normalization. All primer sequences used in this study are detailed in Table S1.

### 2.11. Molecular Docking of DK and DK–Ca with the TRPV6 Channel Key Target Protein

Structural modeling and energy minimization of both DK and DK–Ca were performed using ChemDraw 20.0 and Chem3D 20.0.0.41 software [17]. The crystal structure of the target protein TRPV6 (PDB ID: 6BO8) was downloaded from the PDB database (https://www.rcsb.org/ (accessed on 11 March 2025)). Molecular docking was performed using AutoDock Vina 1.5.7 software following the method of Xue et al. [30] to acquire the optimal conformation. The docking results were analyzed with LigPlot+ 2.2.9 and visualized using PyMOL 2.5.7 and the online database Proteins (https://proteins.plus (accessed on 11 March 2025)).

### 2.12. Statistical Analysis

All assays were conducted in triplicate on three independent biological samples. Univariate ANOVA analysis of the data was performed using BIM SPSS 25.0 software. Tukey’s HSD multiple tests were used for comparison between significant difference groups. *p* < 0.05 was considered statistically significant. Results data were expressed as mean ± standard deviation. The data were graphically created and processed using GraphPad Prism 8 and Origin 2024 software.

## 3. Results and Discussion

### 3.1. Calcium Transport Properties Studies in the Caco-2 Monolayer Model

The integrity of the Caco-2 monolayer model was assessed by measuring changes in the membrane resistance, polarized differentiation, and permeability of the cell monolayer. After 21 days of differentiation, TEER values reached 957 Ω·cm^2^, significantly exceeding the threshold (500 Ω·cm^2^) required for a complete monolayer (Figure S1A), which indicates mature tight junction function. The ALP_AP_/ALP_BL_ activity ratio reached 2.53, confirming established cell polarity (Figure S1B). The apparent permeability coefficient (P_app_) for sodium fluorescein was 8.35 ± 0.15 × 10^−7^ cm/s after 120 min, which is below the threshold (1 × 10^−6^ cm/s) required for tight junction integrity (Table S2). These parameters validated that the Caco-2 monolayer model was functionally intact and appropriate for intestinal calcium transport studies.

#### 3.1.1. The Concentration Selection of DK and DK–Ca

**Figure 1:**
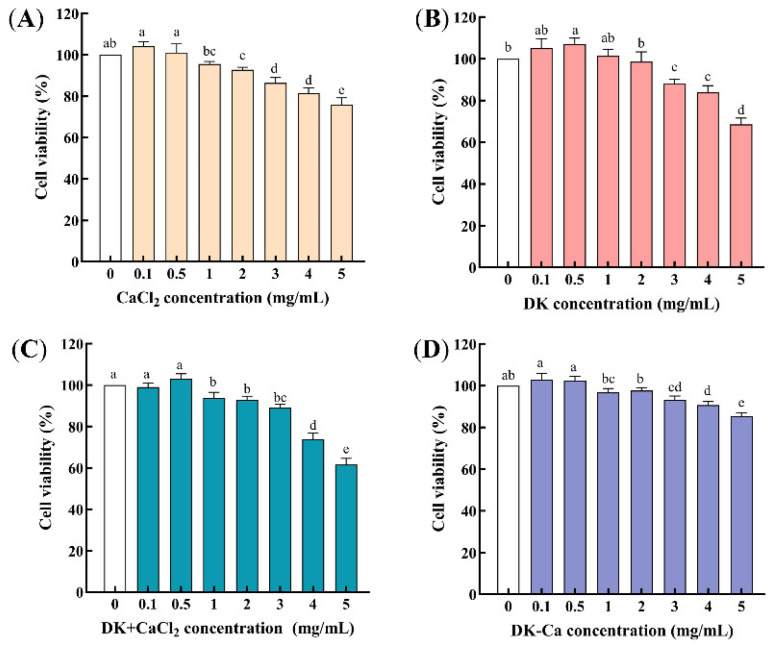
(**A**) Effect of different concentrations of CaCl_2_ on the cell viability of Caco-2 cells; (**B**) Effect of different concentrations of DEEENDQVK (DK) on the cell viability of Caco-2 cells; (**C**) Effect of different concentrations of DEEENDQVK + CaCl_2_ (DK + CaCl_2_) on the cell viability of Caco-2 cells; (**D**) Effect of different concentrations of DEEENDQVK–calcium complex (DK–Ca) on the cell viability of Caco-2 cells. Different lowercase letters indicate significant differences among different concentrations (*p* < 0.05, *n* = 3).

Cell cytotoxicity was evaluated using the MTT assay, with samples considered cytotoxic if the cell viability dropped below 90% at the tested concentration [21]. As shown in Figure 1, after treating Caco-2 cells with each sample for 24 h, the cell viability exceeded 90% for CaCl_2_, DK, and DK + CaCl_2_ within the concentration range of 0 to 2 mg/mL, while that of DK–Ca remained effective even at 4 mg/mL. This indicates that there is no cytotoxicity within this range, and that it is suitable for subsequent experiments.

#### 3.1.2. The Concentration and Time Selection of Calcium Transport

**Figure 2:**
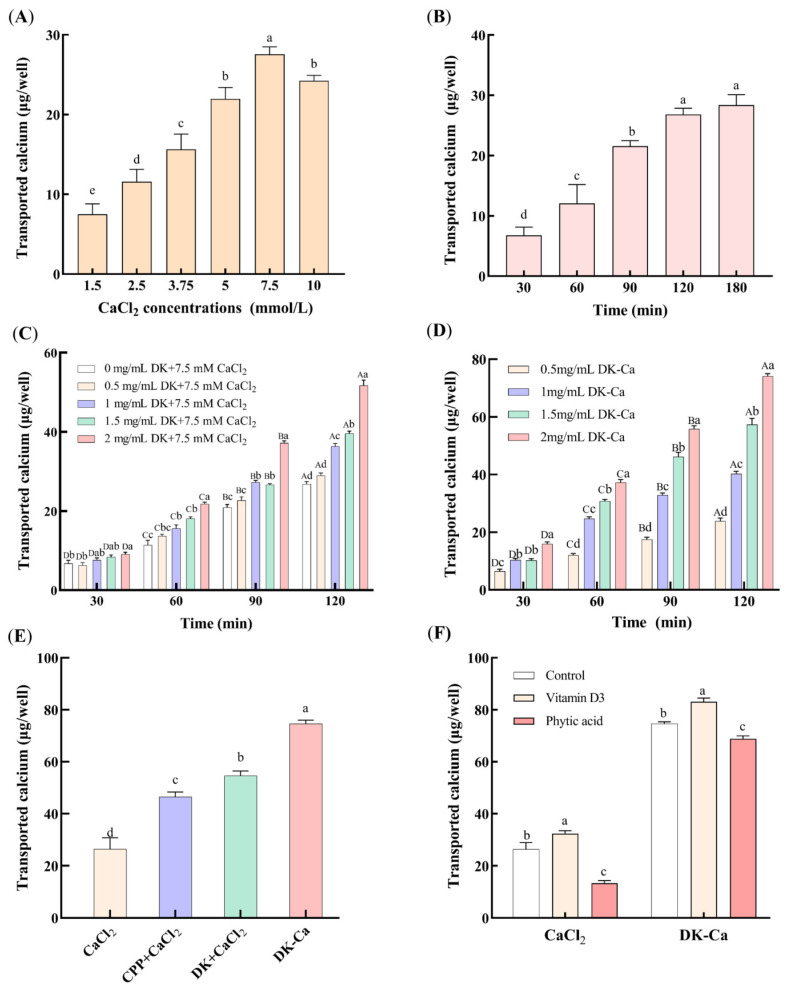
Effect of various factors on transported calcium. (**A**) CaCl_2_ concentration, (**B**) CaCl_2_ transport time, (**C**) different concentrations of DK + 7.5 mM CaCl_2_ during calcium transport-promoting process, (**D**) different concentrations of DK–Ca during calcium transport-promoting process, (**E**) different samples, (**F**) different dietary components. The control group contained only CaCl_2_ or DK–Ca, without addition of vitamin D3 and phytic acid. Different lowercase letters indicate significant differences among different groups (*p* < 0.05, *n* = 3). For (**C**) and (**D**), different lowercase letters indicate significant differences among different concentrations at the same time point (*p* < 0.05, *n* = 3); different uppercase letters indicate statistically significant differences among different time points at the same concentration (*p* < 0.05, *n* = 3).

To evaluate the suitability of the Caco-2 monolayer model for calcium transport studies, determining the optimal concentration and time for calcium transport was essential. As shown in Figure 2A, the transported calcium increased dose-dependently within the concentration range of 1.5~7.5 mM, reaching a maximum of 27.55 ± 1.20 μg/well at 7.5 mM. Further increasing the calcium concentration significantly reduced the transported calcium. This reduction may be attributed to the saturation limits of key active transport proteins, including calbindin-D9k, PMCA1b, and NCX1. When the extracellular calcium concentration exceeds the transport protein binding threshold, the active transport pathway reaches saturation, which leads to a decrease in the calcium transport rate per unit of time [31]. Consequently, a 7.5 mM concentration was chosen for subsequent experiments. Figure 2B shows that, under a calcium concentration of 7.5 mM, the transported calcium increased significantly within 120 min and subsequently remained stable. Given the typical 2~3 h intestinal transit time of food in humans and the need to maintain Caco-2 monolayer integrity, 120 min was chosen as the optimal time for calcium transport assays.

#### 3.1.3. Calcium Transport-Promoting Properties of DK and DK–Ca

Figure 2C presents the effects of different DK concentrations on the transported calcium during the calcium-promoting transport process in the Caco-2 monolayer model. The transported calcium of DK at the same concentration increased significantly with the incubation time in all groups (*p* < 0.001). At 0.5 mg/mL, the DK exhibited no significant difference compared to the CaCl_2_ control (without DK) (*p* > 0.05, 30 min: *p* = 0.596, 60 min: *p* = 0.064, 90 min: *p* = 0.070, 120 min: *p* = 0.099), which indicates that 0.5 mg/mL of DK had no calcium transport-promoting activity. With 1~2 mg/mL of DK, the transported calcium did not differ significantly from the control within the first 30 min. However, from 60 to 90 min, 1~2 mg/mL of DK significantly enhanced the transported calcium compared to the control (*p* < 0.001). After 120 min, the transported calcium increased dose-dependently with the DK concentration (*p* < 0.001), reaching a maximum (51.70 ± 1.91 μg/well) at 2 mg/mL of DK. Xu et al. [32] also showed that the mussel-derived peptide IEELEEELEAER promoted calcium transport in a concentration-dependent manner. Similarly, Liao et al. [13] observed that the casein-derived calcium-binding peptide VLPVPQK promoted calcium transport in the Caco-2 monolayer. Cao et al. [33] further reported that the P5 component from casein phosphopeptides (CPPs) enhanced calcium transport by 4.0-fold compared to CPP-free controls after 240 min. The calcium transport-promoting property of DK observed in this study is consistent with the above findings, which indicates its efficacy in promoting calcium transport in the Caco-2 monolayer model.

Figure 2D presents the impacts of different DK–Ca concentrations over time on calcium transport. Within the same DK–Ca concentration group, the transported calcium showed a significant increase with longer incubation times (*p* < 0.001). The transported calcium increased dose-dependently with the DK–Ca concentration throughout all transport times (*p* < 0.001), except for the initial 30 min. After 120 min, the transported calcium at 2 mg/mL of DK–Ca (calcium content of 300 μg/mL, equivalent to 7.5 mM CaCl_2_) reached 74.11 ± 1.25 μg/well. Figure 2E compares the transported calcium of different samples after transporting for 120 min. The transported calcium of CPP + CaCl_2_, DK + CaCl_2_, and DK–Ca was 0.76-fold, 1.07-fold, and 1.83-fold higher than that of the CaCl_2_ control group, respectively. The result demonstrates that DK–Ca and DK exhibit a stronger calcium transport-promoting activity than CPP and CaCl_2_, with DK–Ca showing superior efficacy. These findings indicate that DK–Ca effectively promotes calcium transport at the cellular level.

This study’s results align with earlier findings regarding peptide–calcium complexes. Yang et al. [34] reported that the rice protein-derived AHVGMSGEEPE (AHV) had a calcium-binding capacity of 55.69 mg/g; its complex (AHV-Ca) promoted calcium transport in Caco-2 monolayers, reaching 61.75 ± 13.23 μg/well (2.16-fold versus CaCl_2_). Similarly, Cui et al. [35] found that the sea cucumber egg-derived NDEELNK–calcium complex exhibited superior calcium transport activity compared to CaCl_2_. Sun et al. [36] demonstrated that the egg white hydrolysate-derived DHTKE–calcium complex increased the transported calcium by seven-fold compared to the calcium-free control group. Notably, these peptides share structural similarities with DK, which are characterized by the presence of Asp, Glu, or His residues, which are critical for calcium binding and transport promotion [36].

#### 3.1.4. Influence of Dietary Components on Calcium Transport

Dietary components such as vitamin D3 promote calcium absorption, whereas phytic acid and oxalic acid inhibit absorption by forming calcium precipitates. This study examined the influence of vitamin D3 and phytic acid on calcium transport mediated by CaCl_2_ and DK–Ca. As shown in Figure 2F, vitamin D3 significantly increased the transported calcium for both the CaCl_2_ and DK–Ca groups (*p* < 0.05, CaCl_2_: *p* = 0.046, DK–Ca: *p* = 0.002), by 22.68% and 11.3%, respectively, compared to the control group. This result demonstrated that vitamin D3 was involved in the regulation of calcium-promoting transport by CaCl_2_ or DK–Ca. Currently, numerous studies have shown that TRPV6 channels are regulated by vitamin D3. Meyer et al. [37] pointed out that TRPV6 is the primary ion channel responsible for calcium entry in the intestinal epithelial cell membrane, and its expression is positively regulated at the transcriptional level by vitamin D3. Kutuzova et al. [38] utilized microarray technology to discover that, in rat small intestines, vitamin D3 can upregulate the expression of intestinal calcium absorption genes, including TRPV6, calbindin-D9k, and PMCA1b, supporting the mechanism by which vitamin D3 induces transcellular calcium transport. Therefore, we speculate that DK–Ca may promote calcium transport through the TRPV6 channel, but further validation is required, such as Western blot analysis. In contrast, phytic acid significantly reduced the transported calcium for the CaCl_2_ and DK–Ca groups (*p* < 0.05, CaCl_2_: *p* = 0.02, DK–Ca: *p* = 0.012), by 49.11% and 7.83%, respectively, compared to the control group. Compared to DK–Ca, phytic acid demonstrated significantly stronger inhibition on calcium-promoting CaCl_2_ transport, which indicates that DK–Ca could resist phytic acid-mediated calcium inhibition and promote cellular calcium absorption. This might be because DK has a superior calcium-binding capacity compared with phytic acid, which helps maintain intestinal calcium solubility. This result was consistent with studies on other peptide–calcium complexes. Lin et al. [39] reported that phytic acid significantly decreased the calcium absorption for CaCl_2_ by 79.86%, while the snapper fish scales protein hydrolysate–calcium complex (FSPH–Ca) could resist the inhibitory effect of phytic acid on calcium, reducing the inhibition of calcium absorption by 21.55%. Similarly, Zheng et al. [2] demonstrated that the walnut peptide–calcium complex (WP-Ca) resisted calcium precipitation caused by phytic acid, maintaining the calcium bioavailability as unchanged, while the CaCl_2_ group significantly decreased the calcium precipitation.

#### 3.1.5. Component Changes in DK–Ca Calcium Transport Promoting in the Caco-2 Monolayer

**Figure 3:**
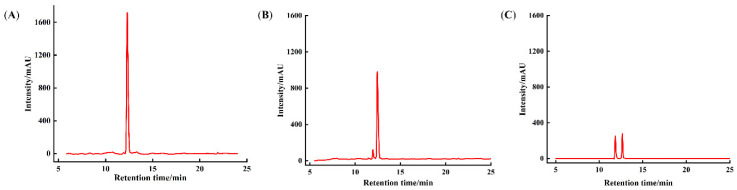
Component changes in DK–Ca calcium transport promoting in the Caco-2 monolayer. (**A**) raw DK–Ca solution, (**B**) solution on the apical (AP) side, (**C**) solution on the basolateral (BL) side.

During Caco-2 cell differentiation, the monolayer model produces brush-border membrane peptidases, which promote the degradation of proteins/peptides into amino acids or shorter peptides [40]. To assess component changes in DK–Ca after 120 min of calcium-promoting transport, solutions collected from the AP and BL sides of the monolayer were subjected to RP-HPLC analysis. As shown in Figure 3, raw DK–Ca showed a single symmetrical peak at 12.55 min, and the peak of DK–Ca existed in both the AP and BL sides. This indicated that a portion of the DK–Ca was transported across the monolayer in its intact form. A portion of the DK–Ca was degraded and produced a new component due to the intensity of the DK–Ca decreasing and a new peak appearing at 11.80 min. Tu et al. [41] found that casein peptides were hydrolyzed by brush-border membrane peptidases in the Caco-2 monolayer and the number of peptide types increased from 121 (7~34 residues) to 184 (7~26 residues). Regazzo et al. [28] demonstrated that the β-casein peptide YQEPVPVRGPFPIIV was cleaved into QEPVPVRGPFPIIV and YQEPVPVRGPFPII after being cultured in Caco-2 monolayers for 120 min. Similarly, the newly appeared peak in our study may correspond to the hydrolysis products from DK–Ca mediated by brush-border membrane peptidases on the AP side. In the future, it will be necessary to identify the degradation products through mass spectrometry analysis.

#### 3.1.6. Visualization of the Calcium Transport-Promoting Effect

**Figure 4:**
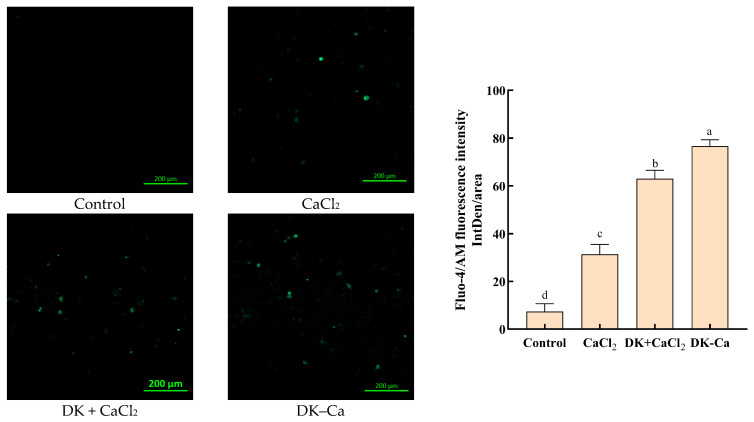
Visualization of the calcium transport-promoting effect. Fluorescence analysis was conducted with a confocal microscope at a magnification of 10×. The exposure time was 0.4 s, the gain was 550 V, and the laser intensity was 1.7%.

To assess the calcium transport-promoting effect, Caco-2 monolayers were treated with different samples and fluorescence imaging based on intracellular calcium influx was examined. The stronger the fluorescence intensity, the higher the intracellular calcium influx [29]. As shown in Figure 4, CaCl_2_, DK + CaCl_2_, and DK–Ca groups significantly increased the fluorescence intensity compared to the control group. Moreover, the order of strength of the calcium-promoting transport effect is as follows: DK–Ca > DK + CaCl_2_ > CaCl_2_. This indicated that DK–Ca can enhance the bioavailability of calcium. Similarly, the egg white-derived DHTKE–calcium complex [36], CPP–calcium complex [42], and soy protein hydrolysate–calcium complex [43] promote intracellular calcium influx, demonstrating the broader applicability of peptide–calcium complexes in improving calcium absorption.

### 3.2. Investigation of the Calcium Transport Pathway

#### 3.2.1. The Concentration Selection of Channel Regulators

**Figure 5:**
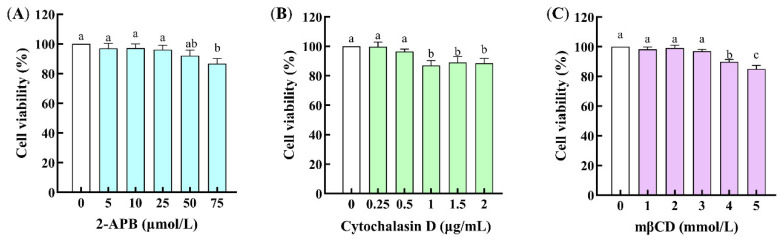
Effects of the channel regulators on the cell viability of Caco-2 cells. (**A**) 2-Aminoethoxydiphenyl borate (2-APB); (**B**) cytochalasin D; (**C**) methyl-β-cyclodextrin (mβCD). Different lowercase letters indicate statistically significant differences (*p* < 0.05, *n* = 3).

The cell cytotoxicity of the channel regulators was assessed by the MTT method. As illustrated in Figure 5, 2-APB in the concentration range of 0~50 μmol/L, cytochalasin D in the range of 0~0.5 μg/mL, and mβCD in the range of 0~3 mmol/L have no cytotoxicity to Caco-2 cells. Thus, 50 μmol/L of 2-APB, 0.5 μg/mL of cytochalasin D, and 3 mmol/L of mβCD were suitable for the following experiments.

#### 3.2.2. Calcium Transport-Promoting Pathways of DK and DK–Ca

**Figure 6:**
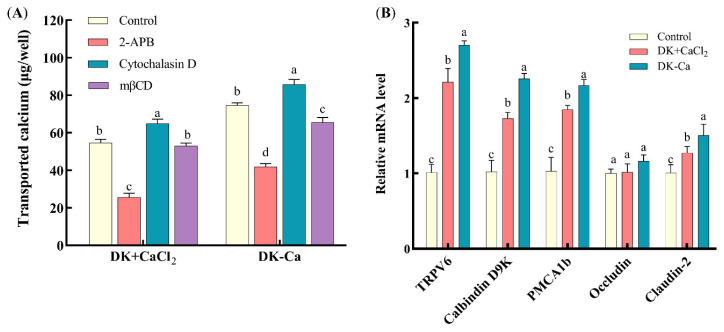
(**A**) Effects of different channel regulators on transported calcium. Different lowercase letters indicate significant differences among channel regulators within the same sample group (*p* < 0.05, *n* = 3). (**B**) Effects of different samples treated with Caco-2 monolayers for 24 h on the expression of calcium channel-related genes. Different lowercase letters indicate statistically significant differences among samples within the same gene (*p* < 0.05, *n* = 3).

To explore the calcium transport-promoting pathways of DK and DK–Ca across the Caco-2 monolayer, transported calcium was measured under the treatment of specific calcium channel regulators. The inhibitor 2-APB elicits conformational changes in TRPV6 channels, consequently inhibiting calcium transport [44]. Cytochalasin D dilates the intercellular spaces, compromising tight junction integrity and thereby augmenting paracellular calcium transport [34]. mβCD predominantly inhibits cholesterol-dependent endocytosis through the extraction of membrane cholesterol, thus perturbing physical characteristics and the functional architecture of the membrane [45]. As shown in Figure 6A, for the DK + CaCl_2_ group, compared to the control group, 2-APB treatment reduced the transported calcium by 53.90% and cytochalasin D treatment improved the transported calcium by 18.5%, while mβCD treatment had no significant change. This result demonstrated that DK promoted calcium transport predominantly through the TRPV6 channel and secondarily through the paracellular pathway, but not through the endocytic pathway. For the DK–Ca group, compared to the control group, 2-APB and mβCD treatment reduced the transported calcium by 43.94% and 12.35%, while cytochalasin D treatment improved the transported calcium by 14.82%. This result indicated that DK–Ca promoted calcium transport predominantly through the TRPV6 channel, with supplementary contributions from paracellular and endocytosis channels. Yang et al. [34] reported that the rice peptide–calcium complex (AHVGMSGEEPE-Ca) might promote calcium transport through the transcellular channel, not through the paracellular and endocytosis channels. Chen et al. [26] demonstrated that cod skin-derived polypeptide GPAGPHGPPG–calcium complex promotes calcium transport through the endocytosis channel. The above studies indicated that different bioactive peptides might promote calcium transport through different pathways.

DK–Ca is a complex formed by chemically chelating DK with CaCl_2_, in which calcium exists in a chelated state. This means the calcium is stably bound to DK via coordinate bonds. This chelated form of calcium exhibits greater stability in the intestinal environment and is less susceptible to precipitation due to dietary components such as stomach acid and phytic acid [9]. DK + CaCl_2_, in contrast, is a mixture of DK and CaCl_2_, where calcium chloride serves as a typical ionic calcium source. Upon dissolution in water, it completely dissociates into free Ca^2+^. Absorption of ionic calcium occurs via active transport (e.g., TRPV6 channels) or passive diffusion [46]. This form of calcium is significantly affected by pH and competitive inhibition from other dietary components, such as phytic acid and oxalic acid [47]. These two forms of calcium are fundamentally different, which may influence their transport processes.

This study examined the transport capacity (Figure 2E) and transport pathways (Figure 6A) of the two calcium forms at the same concentration (7.5 mM). The results showed that, under equivalent dose conditions, the calcium transport-promoting capacity of DK–Ca was 1.37 times that of DK + CaCl_2_. This indicates that chelated calcium has a higher transport capacity than ionic calcium. Similarly, Li et al. [22] used a Caco-2 monolayer cell model to compare the bioavailability of CPP + CaCl_2_ and CPP–Ca at equivalent calcium concentrations. Their results showed that the bioavailability of CPP–Ca (52.03%) was significantly higher than that of CPP + CaCl_2_ (40.29%). This indicates that calcium in the form of chelated peptides is more effective in promoting calcium absorption. Furthermore, although both DK + CaCl_2_ and DK–Ca primarily promote calcium transport through the TRPV6 channel, the calcium transport pathway of DK + CaCl_2_ differs from that of DK–Ca in not involving endocytosis. This difference likely arises because DK–Ca can be transported intact via the endocytosis pathway, while, in the DK + CaCl_2_ group, calcium ions primarily exist as free ions and are transported across the membrane via ion channels and transport proteins, and thus do not rely on endocytosis.

### 3.3. Mechanism of DK and DK–Ca in Calcium Transport-Promoting

#### 3.3.1. Expression Levels of Calcium Channel-Related Protein Genes

To study the mechanisms of DK and DK–Ca in promoting calcium transport, the mRNA expression levels related to calcium transport channels (TRPV6, calbindin-D9k, PMCA1b, occludin, and claudin-2) were quantified. As illustrated in Figure 6B, compared to the untreated control group, DK–Ca and DK + CaCl_2_ treatments significantly upregulated TRPV6, calbindin-D9k, and PMCA1b mRNA expression by 2.70-fold and 2.21-fold, 2.26-fold and 1.73-fold, and 2.17-fold and 1.85-fold, respectively. These results suggest that DK–Ca and DK + CaCl_2_ may promote calcium absorption through TRPV6 channels. When calcium enters the TRPV6 channel, it binds to calbindin-D9k, facilitating the transport of calcium into the cytoplasm and ultimately promoting its release into the bloodstream via PMCA1b. However, further research, such as Western blot analysis, is necessary to determine whether these transcriptional changes result in corresponding increases in the protein levels and functional activities of these transporters. Lin et al. [39] demonstrated that fish scale protein hydrolysate (FSPH) chelated calcium and promoted calcium transport through the TRPV6 channel, while FSPH–Ca upregulated TRPV6, calbindin-D9k, and PMCA1b mRNA expression. Similarly, Li et al. [22] demonstrated that CPP–Ca and CPP + CaCl_2_ regulated TRPV5 and TRPV6 mRNA expression, while CPP–Ca specifically upregulated calbindin-D9k and PMCA1b mRNA expression. In addition, compared to the control group, DK–Ca and DK + CaCl_2_ treatments exhibited claudin-2 mRNA expression that was significantly upregulated by 1.50-fold and 1.21-fold, while the occludin mRNA expression remained unchanged. This result indicated that DK–Ca and DK + CaCl_2_ promoted paracellular calcium transport by upregulating claudin-2 mRNA expression. Claudin-2 is critical for forming paracellular cation channels and promoting cation permeability, including that of Ca^2+^. Its upregulation directly promotes paracellular transport capacity, thereby promoting calcium transport [48].

#### 3.3.2. Interaction of DK and DK–Ca withTRPV6 Channel Key Target Protein

Recent studies suggest that dietary calcium supplements can regulate key proteins in the calcium transport pathway, but the precise mechanism remains unclear [34]. Both DK and DK–Ca primarily promoted calcium transport through the TRPV6 channel. Molecular docking was conducted to investigate this mechanism by analyzing the interactions between DK and DK–Ca and the TRPV6 channel protein. The three-dimensional structure of TRPV6 was obtained from the Protein Data Bank (PDB ID: 6BO8) (Figure S2). TRPV6 forms a tetrameric structure consisting of four identical subunits, in which each subunit is composed of six transmembrane domains (S1–S6) and features a hydrophobic region located between S5 and S6 that constitutes the ion channel pore [49]. Notably, a distinct central cavity in the narrower upper half of the TRPV6 protein is exposed to solvent, which is essential for TRPV6′s ion channel functionality [50]. The binding energies of DK–Ca and DK with TRPV6 were −10.0 and −5.5 kcal/mol, respectively, both below −3.0 kcal/mol, which indicates their potential for spontaneous and stable binding to TRPV6 in the natural state [30]. Notably, DK–Ca had a stronger binding affinity for TRPV6 than DK due to its lower binding energy [30], which is consistent with the finding that DK–Ca had stronger calcium transport-promoting activity than DK.

**Figure 7:**
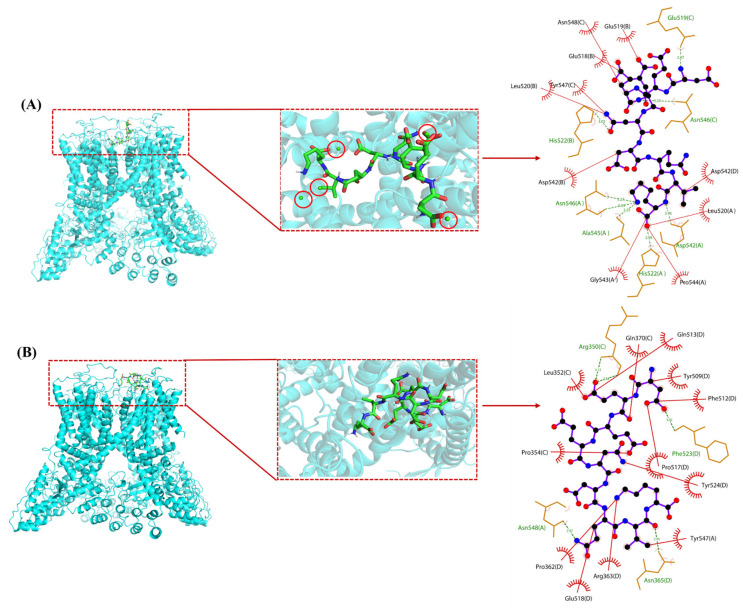
Optimal conformation (**left**) and 2D interaction analysis (**right**) of molecular docking. (**A**) DK–Ca with transient receptor potential vanilloid 6 (TRPV6); (**B**) DK with TRPV6. Green spheres in the red circle represent calcium atoms; black spheres denote carbon atoms; red spheres indicate oxygen atoms; blue spheres stand for nitrogen atoms; green lines symbolize hydrogen bonds; red lines depict hydrophobic interactions.

The molecular docking conformations and interaction sites of DK–Ca and DK with TRPV6 are illustrated in Figure 7. DK–Ca recognized and bound to the active central cavity at the entrance of the TRPV6 channel, increased the local calcium concentration near this area, regulated TRPV6-mediated ion transport, and ultimately promoted calcium transport through TRPV6. DK is bound to the extracellular cavities of TRPV6 formed by S1–S2 and S3–S4 transmembrane domain interfaces. The multiple negatively charged amino acid residues in DK attracted Ca^2+^ toward the channel entrance, facilitating their cellular entry and promoting intestinal calcium absorption. DK–Ca formed 8 hydrogen bonds and 10 hydrophobic interactions with TRPV6, while DK formed 5 hydrogen bonds and 12 hydrophobic interactions. The stronger binding affinity of DK–Ca for TRPV6 suggested that hydrogen bonds played a dominant role in stabilizing the interaction. This result aligned with the findings of Hou et al. [50], who discovered that the calcium-binding peptide derived from duck eggs interacted with TRPV6 via hydrogen bonds and hydrophobic interactions, and had two extracellular binding sites with TRPV6: Site 1 (S5–S6 loop) and Site 2 (S1–S2/S3–S4 cavity).

Molecular docking technology simulates intermolecular interactions to effectively predict the binding patterns of ligand molecules binding to receptor proteins. It is increasingly being applied to explore the mechanisms of action of functional bioactive substances. For example, Xue et al. [30] investigated the inhibitory mechanism of vitelline phosphoprotein peptides (FGTEPDAK and IWGR) on α-amylase activity and insulin resistance using molecular docking and network pharmacology. Wei et al. [51] applied molecular docking to study the antioxidant mechanisms of candida peptides (VPPP and MYP). These studies collectively demonstrate that molecular docking can effectively elucidate the mechanisms of action and potential functions of bioactive compounds. In this study, molecular docking was used to predict the potential binding modes between DK and the TRPV6 protein. The results suggest that DK–Ca enhances calcium uptake through binding to TRPV6. In the future, we will use TRPV6 knockout models and fluorescence resonance energy transfer (FRET) to directly validate the functional involvement of TRPV6 and detect the dynamic interaction between DK–Ca and TRPV6 in live cells.

## 4. Conclusions

This study investigated the calcium transport-promoting properties and mechanisms of DK and DK–Ca using a Caco-2 monolayer model. The order of strength of the calcium-promoting transport effect was as follows: DK–Ca > DK + CaCl_2_ > CPP + CaCl_2_ > CaCl_2_. DK and DK–Ca mainly promoted calcium transport through TRPV6 and paracellular channels by upregulating the mRNA expression of TRPV6, calbindin-D9k, PMCA1b, and claudin-2. DK–Ca bound to the extracellular cavity of TRPV6 through hydrogen bonds and hydrophobic interactions, increased the local calcium concentration at the channel entrance, and facilitated intestinal calcium absorption. As a food-derived peptide-calcium complex, DK–Ca has demonstrated potential for promoting calcium absorption in vitro. However, further animal experiments and clinical studies are still needed to fully clarify the absorption, distribution mechanism, and potential allergenicity of DK–Ca, in order to evaluate its potential application value as a calcium supplement.

## Data Availability

The data presented in this article are available from the corresponding author upon request.

## Supplementary Materials

The following supporting information can be downloaded at: https://www.mdpi.com/article/10.3390/foods14173119/s1, Figure S1: Integrity evaluation of Caco-2 monolayer model; Figure S2:TRPV6 protein 3D structure; Table S1: Primer design; Table S2: Changes in the apparent permeability coefficient of sodium fluorescein in Caco-2 monolayer model.
