# Supplementary material for: Study on the Calcium Transport-Promoting Property and Mechanism of the Peptide–Calcium Complex DEEENDQVK–Ca Based on a Caco-2 Monolayer Model
Source: Foods. 2025 Sep 6;14(17):3119. doi: 10.3390/foods14173119 (PMC12428237; doi:10.3390/foods14173119)
Supplement: Supplementary file 1 [file foods-14-03119-s001.zip › foods-3795481-supplementary.pdf]

Supplementary data

Study on the calcium transport-promoting property and mechanism of the peptide-calcium complex DEEENDQVK-Ca based on a Caco-2 monolayer model

Yaxin Zhang, Jingjing Ru, Shan Gao, Hongli Zhi, Wei Zhao, Chunyan Hao, Xiaowei Zhang*

Tianjin Key Laboratory of Food Quality and Health, College of Food Science and Engineering, Tianjin University of Science & Technology, Tianjin 300457, PR China.

* **Corresponding author.**
E-mail address: zhangxw@tust.edu.cn (Xiaowei Zhang).
Full postal address: No. 29, 13th Road, Tianjin Economic and Technical Development Zone, PR China.

Table S1 Primer design

Analyte	Forward Primer (5’-3’)	Reverse Primer (5’-3’)
GAPDH	TGCACCACCAACTGCTTA	GGATGCAGGGATGATGTTC
TRPV6	ACTGACCTCGACTCTCTATGAC	GTGGTGATGATAAGTTCCAGCAG
CalbindinD9K	CTCCTGAGGAACTGAAGAGGA	TCCATCTCCATTCTTGTCCA
PMCA1b	CTGTTCAGATTGGCAAAGCA	GCAAGTGGAAGACCTTCTGG
Cluadin-2	TATAGCACCCTTCTGGGCCT	GCTACCGCCACTCTGTCTTT
Occludin	ACTTCAGGCAGCCTCGTTAC	CCTGATCCAGTCCTCCTCCA

Table S2 Changes in the apparent permeability coefficient of sodium fluorescein in Caco-2 monolayers model

Time (min)	P_{app} (×10^{-7} cm/s)
30	7.17±0.22 [b]
60	7.47±0.25 [b]
90	7.56±0.64 [b]
120	8.35±0.15 [a]

Note: Different lowercase letters indicate significant differences between data (*P* <0.05).

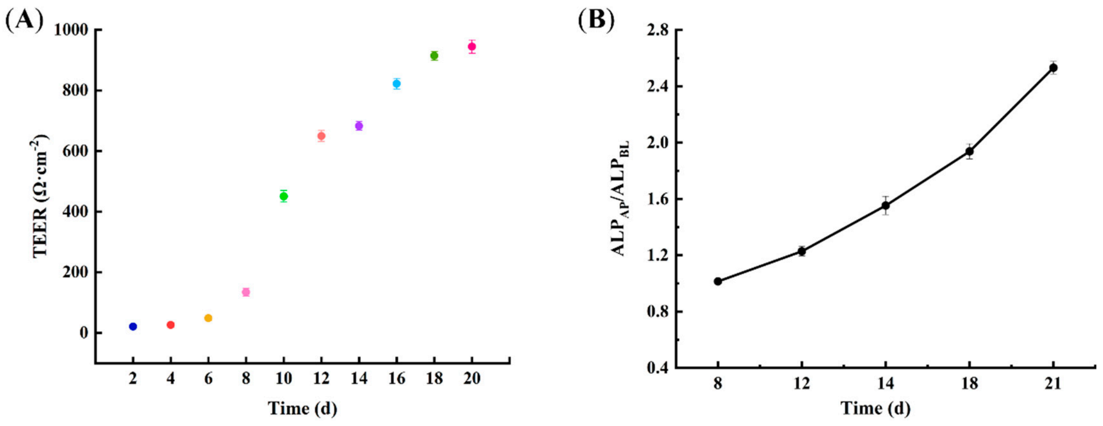

Figure S1 Integrity evaluation of Caco-2 monolayer model. (A) Monolayer membrane resistance values of caco-2 cells. (B) Ratio of ALP activity on both sides.

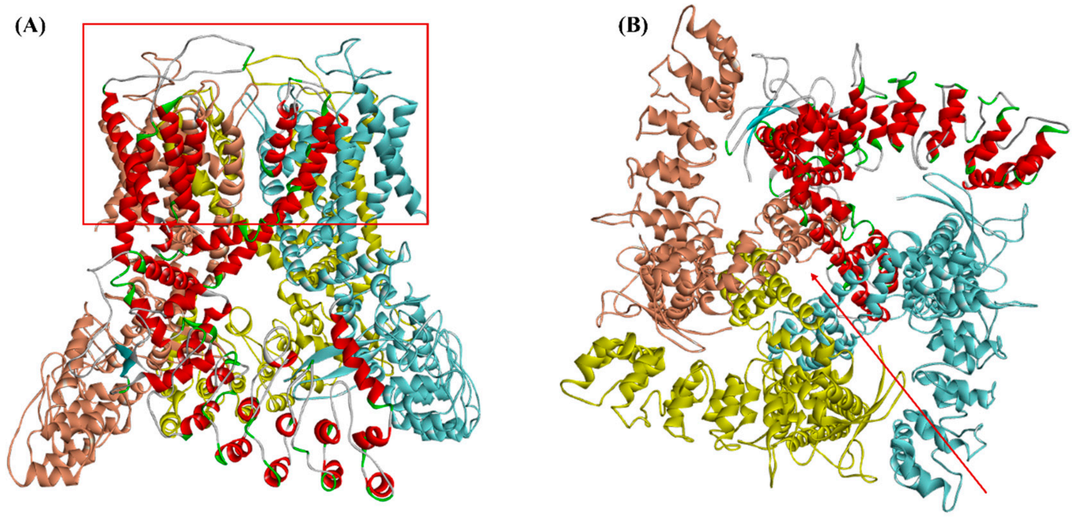

Figure S2 TRPV6 protein 3D structure. (A) plan view; (B) top view.
